# Variation and ethnic inequalities in treatment of common mental disorders before, during and after pregnancy: combined analysis of routine and research data in the Born in Bradford cohort

**DOI:** 10.1186/s12888-016-0805-x

**Published:** 2016-04-12

**Authors:** Stephanie L. Prady, Kate E. Pickett, Simon Gilbody, Emily S. Petherick, Dan Mason, Trevor A. Sheldon, John Wright

**Affiliations:** Department of Health Sciences, University of York, York, YO10 5DD UK; Hull York Medical School, University of York, York, YO10 5DD UK; Bradford Institute for Health Research, Bradford Royal Infirmary, Duckworth Lane, Bradford, BD9 6RJ UK; School of Sport, Exercise and Health Sciences, Loughborough University, Loughborough, LE11 3TU UK

**Keywords:** Anxiety, Depression, Treatment inequality, Ethnic group, Pregnancy, Post-natal

## Abstract

**Background:**

Common mental disorders (CMD) such as anxiety and depression during the maternal period can cause significant morbidity to the mother in addition to disrupting biological, attachment and parenting processes that affect child development. Pharmacological treatment is a first-line option for moderate to severe episodes. Many women prescribed pharmacological treatments cease them during pregnancy but it is unclear to what extent non-pharmacological options are offered as replacement. There are also concerns that treatments offered may not be proportionate to need in minority ethnic groups, but few data exist on treatment disparities in the maternal period. We examined these questions in a multi-ethnic cohort of women with CMD living in Bradford, England before, during and up to one year after pregnancy.

**Methods:**

We searched the primary care records of women enrolled in the Born in Bradford cohort for diagnoses, symptoms, signs (‘*identification’*), referrals for treatment, non-pharmacological and pharmacological treatment and monitoring (‘*treatment’*) related to CMD. Records were linked with maternity data to classify women identified with a CMD as treated prior to, and one year after, delivery. We examined rates and types of treatment during pregnancy, and analysed potential ethnic group differences using adjusted Poisson and multinomial logistic regression models.

**Results:**

We analysed data on 2,234 women with indicators of CMD. Most women were discontinued from pharmacological treatment early in pregnancy, but this was accompanied by recorded access to non-drug treatments in only 15 % at the time of delivery. Fewer minority ethnic women accessed treatments compared to White British women despite minority ethnic women being 55–70 % more likely than White British women to have been identified with anxiety in their medical record.

**Conclusions:**

Very few women who discontinued pharmacological treatment early in their pregnancy were offered other non-pharmacological treatments as replacement, and most appeared to complete their pregnancy untreated. Further investigation is warranted to replicate the finding that minority ethnic women are more likely to be identified as being anxious or having anxiety and understand what causes the variation in access to treatments.

**Electronic supplementary material:**

The online version of this article (doi:10.1186/s12888-016-0805-x) contains supplementary material, which is available to authorized users.

## Background

Common mental disorders (CMD) such as depression and anxiety are chronic, relapsing conditions causing significant morbidity with around one in four adults being affected in one year [1]. While pregnancy and the postnatal years may not be periods of increased prevalence they are sensitive periods, when these disorders can disrupt biological, attachment and parenting processes that subsequently affect child development and behaviour [2, 3].

In general populations, pharmacotherapy and psychological therapies offer effective treatment for CMD, with offer and uptake of specific modalities, or multi-modal treatment, informed by disorder type, current episode severity, past history and patient preference. Treatment with pharmaceuticals is highly prevalent; studies of general (not pregnancy specific) primary-care populations indicating that 63 % of UK patients treated for anxiety and 98 % treated for incident depression receive prescriptions, and in Germany insurance data indicates pharmacotherapy outweighs psychological therapy for patients with major depression by more than 4:1 [4–6]. Pharmacological treatment duration may be considerable - UK guidance recommends antidepressant treatment extends at least six months after remission of symptoms and for a minimum of two years for those at risk of relapse [7], and reviews every 2–3 months are needed to assess the continued need for pharmacological treatment of generalised anxiety and panic disorders [8].

Treatment may be interrupted by pregnancy, when many women discontinue pharmacotherapy [9] for reasons that will likely centre around the perception of potential risk of harm to the foetus. The absolute and relative risks of harm are a subject of controversy, some studies have found a small increased risk of some birth defects associated with SSRI use in early pregnancy e.g. [10], although others have not [11]. Expectant mothers may grossly over-estimate any risks [12], which may mis-inform the decision to discontinue. Both gradual and abrupt discontinuation from antidepressants and benzodiazepines in general populations can result in a variety of temporary distressing withdrawal symptoms, although absolute rates have proven difficult to establish, and there are few studies in pregnancy [13–15]. Of greater clinical concern to the mother and her child is for potential relapse following discontinuation, although little research has been conducted and the two are not conclusively linked [16, 17]. A general increased risk of relapse is associated with greater initial episode severity, and a higher number of previous episodes [16, 17]. There is, however, growing evidence for the negative consequences of untreated CMD during pregnancy for the mother, her developing foetus and the child in later life [18]. Even mild or subclinical disorder causes morbidity and can result in harmful consequences for some offspring of those affected [19]. In the UK, guidance issued to the National Health Service (NHS) promotes access to non-pharmacological treatments for CMD during pregnancy, stepping up with disorder severity to low dose pharmacological treatment, preferably monotherapy, as needed [20, 21]. There are few data to illustrate the extent to which the treatment gap after reduction of pharmacotherapy is filled by other therapies, including psychological treatment.

Rates of common mental disorders (CMD) in the community are higher for those who are socially and economically disadvantaged [22]. People originating from South Asia comprise 5.3 % of the population of England and Wales and, in general, these minority ethnic groups are at higher risk for CMD because they are disproportionally disadvantaged; they may also face additional burden on their mental health due to racism or discrimination [23–26]. Certain sub-groups of ethnic minorities are at higher risk of mental disorder, for example recently migrated South Asian women have around double the burden of distress compared to second or third generation migrants [27].

The Equality Act 2010 decrees that NHS treatment and care should be equitable at point of access, including treatment for common mental disorders. The small number of studies examining CMD treatment disparities for ethnic minorities in the UK and US, however, indicate inequitable prescribing and access to talking therapies [25, 28, 29]. One study in London found variation in the types of drugs prescribed (anxiolytics/hypnotics vs. antidepressants) by ethnicity [30]. There are few published data describing variation in treatment, or under treatment, in the maternal period. This is important to investigate because the higher fertility rate of some minority ethnic women [31, 32] combined with greater risk of mental disorder means that any treatment disparity or variation would have disproportionately large effects on minority ethnic communities.

The Born in Bradford (BiB) birth cohort study provides an ideal data source in which to examine potential minority ethnic inequalities in CMD treatment during pregnancy. Bradford is a city of around 500,000 inhabitants in the North of England with high levels of socio-economic deprivation and ethnic diversity. BiB was set up to examine the impact of environmental, psychological and genetic factors on maternal and child health [33, 34]. Over 12,000 women were recruited during their pregnancy, BiB participants gave permission for their demographic, health and socio-economic data provided at recruitment to be linked with routinely collected sources of data. Previously we have used linked BiB research and primary care data to uncover ethnic disparities in the identification of CMD in primary care [35]. In this study we aimed to examine the quantity and types of treatments offered to women who were identified with CMD before, during and up to one year postnatally, and assess if there was variation in disorders or treatment by ethnic group.

Our research questions were:

1. To what extent is pharmacological treatment curtailed and replaced by non-pharmacological treatment during pregnancy?
2. Do disorder and treatment offered vary by maternal ethnicity?

## Methods

### Population

Women were recruited for the BiB cohort study at the Bradford Royal Infirmary (BRI) between 2007 and 2010, most while waiting for a universally offered glucose tolerance test (GTT) at 26–28 weeks pregnancy [33, 34]. Around 80 % of women attend for their GTT, and >80 % of attendees consented to be recruited into BiB. Most of the enrollees (83 %) filled out a questionnaire reporting socio-demographics, health status and economic situation at recruitment. Enrolled women consented to linkage of routine data and ethics approval for the data collection was granted by Bradford Research Ethics Committee (Ref 07/H1302/112). The enrolled cohort is broadly similar to the pregnant population served by the BRI [34].

### Study data

Data were collected from three sources and linked.

#### Questionnaire at recruitment

We classified self-reported ethnicity into two analytic groups, White British and minority ethnic, based on each woman’s response to questions about ethnic group and cultural background in the recruitment questionnaire. Further decomposition of the minority ethnic group was not practical due to small numbers available for analysis. Self-reported age was obtained from the questionnaire, and we noted which women enrolled subsequent pregnancies into BiB.

#### Maternity database

For women giving birth at the BRI, gestational age at birth and date of birth were obtained from the electronic maternity database and used to calculate date of conception. We performed a simple imputation of gestation as date of birth minus 280 days for the few women who did not give birth at the BRI but whose baby’s date of birth was obtained from summary care records or other NHS sources. We used parity as indicated in the hospital’s maternal record; if this was missing we used the mother’s self-reported parity from the recruitment questionnaire. The first trimester was defined as the conception date through the first 90 days.

#### Primary care records

Nearly all of Bradford’s primary care practices use SystmOne clinical software (© TPP) in which clinical and administrative terms are classified by Read codes, and prescriptions captured using the British National Formulary (BNF) dictionary. SystmOne electronic primary care records were matched to BiB research records by a third party data provider using the NHS number up to February 2013. Matching primary care records were identified for 11,303 (90.8 %) BiB research records.

##### Mental health data

We adapted previously published methods to compile lists of Read codes [36] relevant to the *identification* (signs, symptoms or diagnoses) and *non-pharmacological treatment* (primary care treatment, referrals to secondary and community care, or follow-up) of CMD, and searched records for prescriptions of drugs used to treat CMD, *pharmacological treatment* (Additional file 1: Table S1). We used a sensitive set of Read codes to indicate non-pharmacological treatment, such as GP follow up for a mental health problem, in order to capture events for women where primary treatment or referral may have been recorded just prior to our study period, but treatment took place during the study period. We separated *identification* Read codes into those that indicated depression, or depressive symptoms, and those that indicated anxiety, or anxiety symptoms. We did not have access to free-text notes and referral letters due to third party data protection concerns.

##### Data on further pregnancies

We identified women with further pregnancies that they did not enrol into BiB from primary care records using a Read code search with pregnancy, abortion and foetal death terms, adapting previously published codes [37]. The sensitivity of using the routine data to identify the (known) index pregnancy was 96.0 % (95 % CI: 95.6 to 96.4).

#### Exclusions

We used NHS tracing files, which indicate current area of residence each year, to exclude women who moved from Bradford between enrolment and the end of the first postnatal year. This minimised potential unknown missing data bias caused by some women having incomplete primary care records because they moved to a practice not using SystmOne. We were not able to obtain these data prior to enrolment. We also excluded women with no linked primary care records, with Read codes or prescriptions indicating the possibility of severe mental illness (psychoses, bipolar, schizophrenia), with missing date of birth for the index baby, with a pregnancy in the six-month pre-conception period, who did not fill in the questionnaire at recruitment, who did not provide their ethnicity on the questionnaire, and with missing parity. We excluded occurrences of drugs that had current indications in the BNF for being used to treat medical conditions in addition to CMDs (in this sample this was chlordiazepoxide, doxepin and duloxetine).

#### Included sample

We selected one index pregnancy per mother. If a mother had enrolled multiple pregnancies during the BiB study enrolment period (12,450 women enrolled 13,773 pregnancies), we used the first enrolled pregnancy where she had completed a recruitment questionnaire providing her ethnicity. We used data from all women who, at any time in the period between six months prior to conception and 12 months after the index birth, had markers in the medical record of *identification*, and/or *non-pharmacological treatment*, and/or *pharmacological treatment* of CMD.

#### Analysis periods

To investigate replacement of pharmacological with non-pharmacological treatment we defined a period encompassing the pregnancy. Women were included in this analysis if they had received at least one prescription during pregnancy. We defined two further separate time periods to examine whether treatment varied by ethnicity. The first period spanned from six-months prior to conception up to the birth, the ‘*pre-delivery*’ period, and the second was delivery to 12 months post-delivery; the ‘*first postnatal year’*. Women included in each analysis period were those who were *identified* or *treated* within that period.

#### Derived dependent variables

Derived variables are summarised in Table 1.

**Table 1:** Summary of dependent variables

Variable	Categories
Identification (binary)	Presence of identification Read codes
	Absence of identification Read codes
Identified indication (binary)	Presence of anxiety Read codes
	Presence of depression Read codes only
Identified indication (three-category)	Presence of depression Read codes only
	Presence of anxiety Read codes only
	Presence of comorbid anxiety and depression Read codes, or both anxiety and depression Read codes
Treatment (four-category)	Pharmacological treatment only
	Non-pharmacological treatment only
	Both types of treatment
	No treatment
Pharmacological treatment (three-category	Treated with drugs that are primarily antidepressant
	Treated with drugs that are primarily anxiolytic
	Treated with both types of drugs

Dependent variables were separately derived for each analysis period (pre-birth, postnatal year), see text for further description

##### Identification of CMD

Women included for each analysis period were those with *identification* or *treatment* indicators, however many women only had indicators of treatment (e.g. a prescription, or a referral to a community mental health team) with no corresponding indication of the nature of the problem. Incomplete ascertainment of disorder or problem is common in mental disorder studies that analyse medical records [38, 39]. In order to examine treatments in relation to need, we first classified a binary variable of women being *identified* with CMD in that period.

##### Identified indication

For those women who were identified, we constructed a binary variable of their *indication*, classifying women with an anxiety-related Read code in that period versus women who had depression-related only Read codes. In the interest of parsimony in our small sample, women with co-morbid anxiety and depression disorders, and women who had both anxiety and depression-related Read codes in the same period were classified as having anxiety. We further constructed a three-category indication variable (anxiety, depression and both) in order to examine counts by pharmacological treatment.

##### Treatment for CMD

In order to examine differences in the types of treatments recorded we categorised identified women as having *pharmacological treatment* only, *non*-*pharmacological treatment* only, *both types of treatment*, or *no treatment* in that period in a four-category variable.

##### Pharmacological treatment for CMD

We characterised prescribed drugs as those that were primarily antidepressant and those that were primarily anxiolytic/hypnotic (Table 2) and derived a variable that classified women as being prescribed drugs that were primarily antidepressant, primarily anxiolytic or being prescribed both types of drug in any one period.

**Table 2:** Drug classification

Drug group	Drug class	Drug name
Primarily antidepressant	SSRI	fluoxetine
		citalopram
		escitalopram
		sertraline
		paroxetine
		cipramil
		cipralex
		fluvoxamine
	SNRI	venlafaxine
	TCA	dosulepin
		clomipramine
		imipramine
		lofepramine
	SARI	trazodone
	NaSSA	mirtazapine
Primarily anxiolytic	BETA-B	propranolol
	BENZ	diazepam
		temazepam
		lorazepam
		loprazolam
		oxazepam
		nitrazepam
	non-BENZ HYP	zopiclone
	SED-HYP	zolpidem

Lists all drugs found in medical records spanning the whole study period

#### Covariates

Covariates used in the analyses included maternal age (continuous and quadratic), parity (0,1,2,3,4+), and a further pregnancy in the first postnatal year (binary yes / no). We constructed several binary *indication* and *treatment* covariates. We classified a *depression indication covariate* as the presence or absence of depression indicators in the concurrent period, and, for postnatal analyses, these indicators during the pre-birth period. We similarly constructed an *anxiety indication covariate* for the concurrent and pre-birth periods. A *pharmacological treatment covariate* was the presence or absence of pharmacological treatment in the pre-birth period (used in postnatal analyses); and we similarly defined a non-*pharmacological treatment covariate*.

### Analysis

#### Replacement of pharmacotherapy with other treatments during pregnancy

We assessed the replacement of pharmacological treatment with non-pharmacological treatment during the pregnancy using the denominator of women who had at least one prescription issued during the pregnancy. In this group, we counted the number of women who did not have a prescription issued after the first trimester and had an indication of non-pharmacological treatment in the medical record at any point during the pregnancy. We noted the number of prescriptions by drug group and class. Potential ethnic differences in treatment replacement were not investigated due to small numbers.

#### Variation in treatment by ethnic group

##### Identification of CMD

Potential variation in identification by ethnic group were investigated using Poisson regression, reporting the adjusted relative risk (aRR) for minority ethnic women compared to White British women in each period. We adjusted the pre-delivery analyses for maternal age and parity, and the postnatal analysis for maternal age, parity, further pregnancy, pre-birth depression and anxiety indication covariates and pharmacological and non-pharmacological treatment covariates.

##### Identified indication

For this analysis, we included only women with *identification* Read codes. Potential variation in identification by ethnic group were investigated using Poisson regression on the binary identified indication variable, reporting the aRR for minority ethnic women compared to White British women in each period. We adjusted the pre-delivery analyses for maternal age and parity, and the postnatal analysis for these plus further pregnancy, pre-birth depression and anxiety indication covariates and pharmacological and non-pharmacological treatment covariates.

##### Treatment type

Again including only women with *identification* Read codes we applied multinomial logistic regression to the treatment type variable, holding ‘pharmacological treatment only’ as the reference category and reporting the aRR for minority ethnic women compared to White British women in each period. We adjusted the pre-delivery analyses for maternal age, parity and concurrent depression and anxiety indication covariates, and the postnatal analysis for these plus further pregnancy, and pre-birth depression and anxiety indication covariates. Due to small numbers we were not able to further stratify the analysis by the type of indication (depression / anxiety). Instead we graphed unadjusted proportions of women in each ethnic group who received prescriptions of different groups of drugs by the three-category indication variable.

Post-hoc, we noted a number of prescriptions for propanolol (sometimes prescribed as an anxiolytic), which had, prior to the study, been (until 2007) labelled as a first line anti-hypertensive. To assess whether this affected our results we repeated all regression analyses adjusting for hypertension in pregnancy (the presence or absence of hypertension was ascertained from the electronic maternity system for 88.2 % of women).

We present 95 % confidence intervals (CI) around risk estimates and used Stata release 13 (StataCorp. 2013. Stata Statistical Software: Release 13. College Station, TX: StataCorp LP) to conduct all analyses.

## Results

### Included sample

**Fig. 1:**
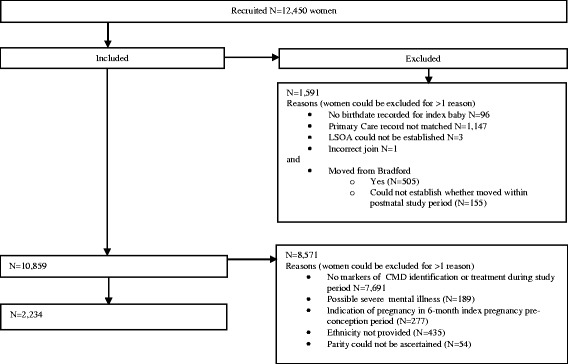
Study flowchart

We included 2,234 women with CMD (as defined in the Methods) during the study period (Fig. 1). Our sample consisted of 1,251 women of White British ethnicity and 983 minority ethnic women (Table 3). We have previously reported that, for similar levels of self-reported psychological distress, minority ethnic women in BiB were less likely to be identified in primary care as having CMD compared to White British women, which we interpret as a primary care identification disparity [35]. Therefore, White British women are over-represented in this analysis (56.0 %) compared to the proportion enrolled in BiB (39.5 %) [34]. Mean age at recruitment for our analysed sample was 26.8 years (standard deviation 5.9). Seventy-six per cent of minority ethnic women and 54 % of White British women lived in areas ranked as the fifth most deprived nationally on the Index of Multiple Deprivation 2010.

**Fig. 2:**
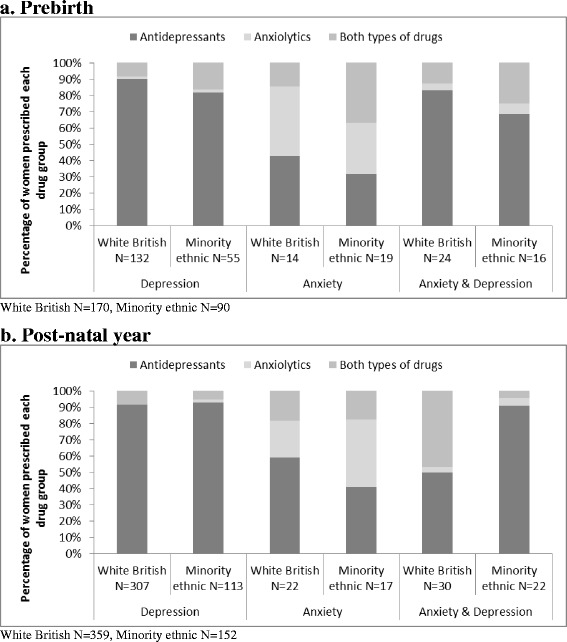
Drug group by indication by ethnic group (**a**) pre-birth (**b**) post-natal year

**Table 3:** Participant characteristics

		White British	Minority ethnic	All
N		1,251	983	2,234
Age at recruitment, mean (SD)		25.9 (6.0)	27.9 (5.6)	26.8 (5.9)
Parity at recruitment, n (%)	First child	614 (49.1)	373 (38.0)	987 (44.2)
	1	369 (29.5)	230 (23.4)	599 (26.8)
	2–3	225 (18.0)	295 (30.0)	520 (23.3)
	4+	43 (3.4)	85 (8.7)	128 (5.7)
Further pregnancy in 12 months postnatal period, n (%)	No	1,149 (91.9)	869 (88.4)	2,018 (90.3)
	Yes	102 (8.2)	114 (11.6)	216 (9.7)
Ethnic group, n (%)	White British	1,251 (100)	-	1,251 (56.0)
	Pakistani	-	749 (76.2)	749 (33.5)
	Mixed	-	64 (6.5)	64 (2.9)
	Indian	-	44 (4.5)	44 (2.0)
	White non-British	-	32 (3.3)	32 (1.4)
	Black	-	27 (2.8)	27 (1.2)
	Bangladeshi	-	26 (2.6)	26 (1.2)
	Other	-	41 (4.2)	41 (1.8)
Country of birth and age at migration, n (%)	Born in UK	1,233 (98.6)	491 (50.0)	1,724 (77.2)
	Immigrated before age 16	13 (1.0)	132 (13.4)	145 (6.5)
	Immigrated 16 or older	2 (0.2)	347 (35.3)	349 (15.6)
	Missing	3 (0.2)	13 (1.3)	16 (0.7)
Language used during recruitment, n (%)	English	1,250 (99.9)	751 (76.3)	2001 (89.6)
	Other language	0	224 (22.8)	224 (10.0)
	Missing	1 (0.1)	8 (0.8)	9 (0.4)
Marital/cohabitation status at recruitment, n (%)	Married & living together	328 (26.2)	791 (80.5)	1,119 (50.1)
	Cohabiting	503 (40.2)	41 (4.2)	544 (24.4)
	Not living with a partner	419 (33.5)	151 (15.4)	570 (25.5)
	Missing	1 (0.1)	0	1 (0.4)
Index of Multiple Deprivation (2010) national rank at recruitment, n (%)	Most deprived	673 (53.8)	751 (76.4)	1,424 (63.7)
	2	253 (20.2)	146 (14.9)	399 (17.9)
	3	220 (17.6)	74 (7.5)	294 (13.2)
	4	75 (6.0)	4 (0.4)	79 (3.5)
	Least deprived	30 (2.4)	8 (0.8)	38 (1.7)

The number of women included in the analyses (i.e. had any Read codes relating to CMD, or prescriptions for treating CMD) in the pre-delivery period was 750 (*N* = 439 White British, 58.5 %; *N* = 311 minority ethnic, 41.5 %) and 1,039 in the postnatal year (*N* = 645 White British, 62.1 %; *N* = 394 minority ethnic, 37.9 %). Fifteen per cent of women (*N* = 335) were included in analyses at both time periods (*N* = 233 White British 69.6 %; *N* = 102 minority ethnic, 30.5 %).

### Replacement of pharmacological with non-pharmacological treatment during pregnancy

During pregnancy, 298 women had one or more prescriptions issued and 144 women had at least one Read code indicating non-pharmacological treatment. Of the 809 prescriptions issued, 69.1 % were for an SSRI (Table 4). Eighty-six per cent of women received an antidepressant and 22.6 % an anxiolytic (some received both). Nearly 60 % (*N* = 174) of the 298 women on pharmacological treatment did not have a prescription dated after the end of the first trimester, when most pregnancy-related pharmacological discontinuation would have taken place. Women who discontinued were just as likely to have been prescribed an antidepressant (86.2 %) and/or anxiolytic (20.7 %) as those who continued. Only 26 of the 174 women with ceased prescriptions (14.9 %; White British 15.1 %, minority ethnic 14.7 %) had a Read code in their medical record indicating provision of a non-pharmacological treatment during the pregnancy.

**Table 4:** Number of prescriptions issued during pregnancy by drug type and class

Drug group	N	%	Drug class	N	%
Primarily antidepressant	689	85.2	SSRI	559	69.1
			SNRI	56	6.9
			TCA	35	4.3
			SARI	26	3.2
			NaSSA	13	1.6
Primarily anxiolytic	120	14.8	BETA-B	31	3.8
			BENZ	39	4.8
			non = BENZ	50	6.2
	809	100		809	100

### Variation by ethnic group

Estimates for regression analyses by ethnic group are presented in Table 5.

**Table 5:** Relative risk of treatment and disorder type by ethnic group

	Pre-delivery					First postnatal year				
	White British *N* = 439		Minority ethnic *N* = 311		Minority ethnic vs. White British	White British *N* = 645		Minority ethnic *N* = 394		Minority ethnic vs. White British
	n	%	n	%	aRR (95 % CI)	n	%	n	%	aRR^1^ (95 % CI)
*Identification Read codes*										
At least one (ref. none)	247	56.3	175	56.3	1.03 (0.85, 1.26)	442	68.5	236	59.9	0.88 (0.75, 1.03)
*Identified indication*	*N* = 247		*N* = 175			*N* = 442		*N* = 236		
Anxiety^a^ (ref. depression only)	67	27.1	83	47.4	**1.69 (1.22, 2.35)**	66	14.9	63	26.7	**1.55 (1.08, 2.23)**
*Treatment type for those identified*					aRR^2^ (95 % CI)					aRR^3^ (95 % CI)
Pharmacological only	98	39.7	58	33.1	ref.	229	51.8	121	51.3	ref.
Non-pharmacological only	22	8.9	16	9.1	1.39 (0.64, 3.02)	14	3.2	14	5.9	2.02 (0.90, 4.54)
Both types of treatment	72	29.2	32	18.3	0.85 (0.49, 1.47)	130	29.4	31	13.1	**0.42 (0.26, 0.68)**
None	55	22.3	69	39.4	**1.96 (1.14, 3.37)**	69	15.6	70	29.7	**1.74 (1.14, 2.69)**

^a^ includes women with comorbid anxiety and depression

aRR Poission regression reporting relative risk adjusted for maternal age and parity

aRR^1^ Poission regression reporting relative risk adjusted for maternal age, parity, anxiety/depression Read codes and treatment type (pharmacological/non-pharmacological) in the pre-delivery period, further pregnancy in the postnatal year

aRR^2^ Multinomial logistic regression reporting relative risk adjusted for maternal age, parity, concurrent anxiety/depression Read codes, further pregnancy in the postnatal year

aRR^3^ Multinomial logistic regression reporting relative risk adjusted for maternal age, parity, concurrent anxiety/depression Read codes, anxiety/depression Read codes and treatment type (pharmacological/non-pharmacological) in the pre-delivery period, further pregnancy in the postnatal year

*CI* confidence interval

bolded estimates are statistically significant

#### Identification

Fifty-six percent of women had at least one identification Read code in the pre-delivery period and 65 % in the first postnatal year. Similar proportions of women in each ethnic group had identification Read codes.

#### Identified indication

Minority ethnic women were 69 % more likely to have indications of anxiety rather than depression in the pre-delivery period compared to White British women (similarly 55 % more likely in the first postnatal year).

#### Treatment type for those identified

In the pre-birth period, holding pharmacological treatment only as the reference category, compared to White British women, minority ethnic women were nearly twice as likely to have no treatments recorded. A similar result was observed when analysing data from the first postnatal year, where additionally, minority ethnic women were less than half as likely to have both pharmacological and non-pharmacological treatment. Very few women in either period had non-pharmacological treatment only. All results were robust to adjusting for hypertension during pregnancy (data not shown).

#### Drug group by indication

Visual inspection of unadjusted counts of drug group/s prescribed by indication revealed little variation by ethnic group (Fig. 2).

## Discussion

### Summary of findings

We analysed routinely collected primary care data from 2,234 maternal women with markers of common mental disorders living in an ethnically diverse and economically deprived city. The majority of women were discontinued from pharmacologic treatment prior to and during pregnancy but this did not appear to be balanced by an increase in access to non-drug treatment such as psychological therapies. Minority ethnic women were more likely than White British women to have a marker of diagnosis, symptoms or signs of anxiety rather than depression in their medical record. Adjusted for indication, minority ethnic women appeared to have less access to treatment both pre- and post-birth, and were less likely to be recorded as being treated with both pharmacology and non-pharmacological modalities in the postnatal year than White British women.

### Strengths and limitations

This study included a large number of minority ethnic women with accurate data on gestation dates and further pregnancies. We used a broad definition of CMD to minimise recording variation in the medical record. Research data were linked to the primary care record using unique identifiers, minimising differences in quality of non-unique personal identifiers between ethnic groups. The tight geographic focus minimises potential bias due to regional variation in coding or prescribing practices which could be confounded with ethnic density in different regions. This study demonstrates the power of data linkage to address important health services research questions and identify areas for quality improvement.

Our study has several limitations. There was some overlap in women included in analyses for both time periods which may have affected our estimates. Due to small numbers we were not able to sensibly analyse the data by more precisely defined ethnic groups, which will have led to potentially substantial heterogeneity within our categorised groups. We had no access to medical records prior to six-months pre-conception which will have led to an under-estimate of treatments if referrals or prescriptions were made just before this date, or identification was noted prior to this date. Additionally, we did not have access to the contents of referral letters or free-text notes which may have led to under-estimates, or the misrepresentation of inequalities, if these data were systematically different by ethnic group. The recognition and management of CMDs by midwives or health visitors may not have been recorded in the electronic record, but because GPs should be notified of suspected CMD [20, 21], we do not think this impacted materially on our results.

For classifying women as ‘*identified*’ we did not rely on Read codes indicating diagnoses because they have only 6–26 % sensitivity for disorder [38, 39], recording of diagnoses versus symptoms appears to change over time [38, 40], and we were unsure whether either of these factors also varied by practice. We had no indication of illness severity, and combining symptoms and signs with diagnoses may have hampered our analysis of whether and with what ‘*identified’* women were ‘*treated*’ if this varied by ethnic group. Our Read code list, while extensive, may not be exhaustive. By including Read codes for community referrals and monitoring appointments we operationalised a rather liberal definition of non-pharmacological treatment which may include women who were not in receipt of an effective treatment. Our definition of ‘*treatment*’ will overestimate the number of women actually undergoing treatment because we did not know which women started, completed, or dropped out of treatment. This means actual treatment rates are lower than reported here. In line with other estimates of general and maternal primary care attendees [39, 41, 42], treatment rates in this cohort under-represent the number in potential need with psychological distress undetected in primary care for an estimated 31–47 % of women [35]. Because minority ethnic women are more than twice as likely to have an unrecognised disorder and therefore no NHS treatment [35], our results are conservative and under-represent absolute disparity in service use and treatment need. Our estimates are of event coding in the medical record and not treatment uptake or success, neither are they adjusted for need, which may vary considerably between and within ethnic groups. The lack of a reliable indicator in primary care of disorder severity or amelioration meant we could not differentiate between women who were discontinued from pharmacotherapy because they no longer needed treatment, and those with active disorder requiring further support. This may have affected our findings if discontinuation by indication varied between ethnic groups.

Other factors such as education or ability in English may influence treatment sought or received, such as access to psychological therapies, but it was beyond the scope of our descriptive study with small numbers of treated women to further stratify our analyses. As with all analyses of routine general practice in the UK there are no linked data to indicate which prescriptions were filled, or which courses were completed, and we did not have diagnostic data available which would have enabled more accurate estimation of treatment inequalities. We did not have access to information about non-NHS treatments, such as private counselling, which may vary between ethnic and socio-economic group. Finally, the cohort is based in one city and results may not be generalisable to other areas, or nationally.

### Treatment during pregnancy

Our study agrees with other data from the UK, Nordic countries and the Netherlands showing that most women taking antidepressants prior to or at the start of pregnancy discontinue them before delivery [9, 43–45]. Our results add to this literature by demonstrating that, for most women, withdrawal from pharmacological treatment does not seem to be replaced with alternative active treatments. The implication is that a proportion of women with active anxiety or depression symptoms are simply waiting out their pregnancy without treatment. UK guidance covering the study period recommended that after withdrawal of pharmacological treatment of mild depression before or during pregnancy, doctors should monitor symptoms or consider referral for brief psychological treatment [20]. New guidance brought in after the end of our study period recommend facilitated self-help in these cases [21]. For those experiencing moderate or severe depressive episodes, and those with anxiety, recommendations were to switch drugs to those with a better safety profile, or switch to a psychological treatment [20]. These recommendations are unchanged in the new guidance, with increased emphasis on the management of anxiety disorders in pregnancy, for which non-pharmacological treatment is the preferred option [21].

Assuming all those in our sample who were treated pharmacologically had CMD, that depression accounted for 50 % of diagnoses, and that 70 % of depression cases were mild [46], then 65 % of women had either anxiety, or a moderate to severe depressive episode that guidelines suggest should have remained on treatment. Our results indicate that only 15 % of women who discontinued were offered non-pharmacological treatment by the end of gestation, but women in active treatment at any one time will have been lower, as we could not adjust for cessation of time-limited psychological therapy, time lag between referral and uptake, or take up rate. It seems unlikely that 85 % of women discontinuing pharmacotherapy during the first trimester had ameliorated symptoms that did not require any further treatment. Rates of replacement therapy do not appear to approach the potential need and raise serious questions about increased risk of morbidity to the mother, the foetus and the family. During the study period the Improved Access to Psychological Therapy (IAPT) programme was piloted and then rolled out in England by the Department of Health with an aim of equitably treating people with CMD using evidence-based talking therapies (largely cognitive behavioural therapy but also including interpersonal and brief dynamic interpersonal psychotherapy, and counselling and couples therapy for depression) [47, 48]. The IAPT programme aims to treat 15 % of CMD prevalent in the community, or, allowing for patient choice, 72 % of those identified in primary care [48]. In time this programme has the potential to close this apparent treatment gap, however, demand currently outstrips provision, leading to long waiting times [48] that might outlast the pregnancy for some. Initial analyses indicate potential disparity in uptake and outcomes [49] and acceptability and local availability of non-pharmacological treatment as potential sources of health inequality requires further investigation, particularly in respect to cultural adaptation of interventions for South Asian women and non-English speakers [50]. Further research into treatment decision-making in primary care is needed, including training and education gaps [51].

### Treatment inequalities

National studies utilising prescription data (without accounting for specific disorders) have found associations between increased prescribing of anxiolytics/hypnotics and antidepressants in practices based in areas of high deprivation, and some evidence for decreased prescribing in areas with greater proportions of Asian, Asian British or minority ethnic patients, and in single-handed practices [52–55]. As minority ethnic women are more likely to live in areas of higher deprivation in the Bradford district, they may encounter dual inequalities linked to both cultural and geographic factors.

Our finding of potential treatment variation for minority ethnic (mostly Pakistani) women adds information about the maternal period to, and broadly concurs with, other, non-maternal, studies on minority ethnic inequalities. Our denominator only included primary care attendees with markers in their medical record that are indicative of CMD. When inequalities related to primary care detection rates are factored in, the risks of under-treatment for ethnic minorities in the community will be higher [28, 35, 56]. While our analysis did not uncover gross prescribing differences, small numbers and a lack of precision may have hampered our analysis. In an English nationally representative study Cooper and colleagues found that, after controlling for symptom severity, White people were twice as likely to be prescribed antidepressants for CMD compared to Black people [28]. Small numbers hampered analysis in South Asian ethnic groups and anxiolytics were not studied. Gater and colleagues in a study based in the city of Manchester found that White European women who were depressed, according to a diagnostic interview outside of the primary care setting, were twice as likely to have medical records that indicated antidepressant prescriptions (50 % vs 23 %) and four times more likely (25 % vs 6 %) to have been given psychological treatment than depressed Pakistani women [25]. Ecological data from East London found that practices with high numbers of Asian people on their lists prescribed slightly *more* antidepressants compared to anxiolytics/hypnotics [30].

Our finding that minority ethnic women were more likely to be identified with anxiety/report anxiety symptoms than depression/depressive symptoms compared with White British women is new, but the implications difficult to interpret without more accurate diagnosis information. It could indicate a true difference in specific disorder prevalence by ethnicity in this sample. If so, because treatments for anxiety are primarily psychological, we would expect more minority ethnic women to be accessing non-pharmacological treatments, which they did not. This may indicate the under-provision of culturally adapted psychologically-based treatments. Although small numbers prevented us exploring the relationship between disorder type and specific treatments further, our results may indicate increased prescription of anxiolytics for need, which is a concern in view of the long recognised risks of tolerance and dependence. Alternatively, our finding may point to over-characterisation of minority ethnic women with anxiety rather than depression, along with potentially increased levels of inappropriate prescription of anxiolytics for depressive symptoms. There is little other evidence on specific disorder prevalence for different ethnic groups, but one study of a nationally representative sample did not detect statistically significant variation [24]. Long standing questions over the validity of cross-cultural measurements and differential accuracy of diagnoses still hinder our understanding in this area [57, 58]. Further research using larger samples, longitudinal data and mixed methods are needed to replicate these and other findings and unpick whether there are differences in need (i.e. severity and type of illness varies by ethnic group), variation in Read coding by patient (or provider) ethnic group, systematic differences in preference whereby certain treatments are less attractive to minority ethnic women, or whether there is variation in the type of treatments offered to women of different ethnic groups by GPs.

The dearth of research into maternal ethnic mental health disparities translates to a lack of specific advice to primary care healthcare practitioners, who identify and treat the majority of CMD cases, on how to ensure that identification and treatment choices are equitable. Even where advice is clear, the gap between best and actual practice may be large [59]. Primary and community care staff need support, including better data, to enable them understand the impact of mental health inequalities on their patients and how to address them [60].

## Conclusions

We found evidence of mental health variation and potential disparities for maternal minority ethnic women that warrant further investigation to close health inequality gaps. While most women are discontinued from pharmacological treatment during pregnancy according to treatment guidelines, the seeming lack of provision of alternative active treatments is concerning and may have significant future negative effects for families.

### Ethics approval and consent to participate

Ethics approval for the data collection was granted by Bradford Research Ethics Committee (Ref 07/H1302/112).

### Consent for publication

Not Applicable.

### Availability of data

Requests to analyse Born in Bradford data are reviewed on a monthly basis by the BiB Executive Group. The BiB website (www.borninbradford.nhs.uk) has details of how to submit requests for access to raw data.

## Additional file

Additional file 1: Table S1. List of drug prescriptions and Read codes. (DOCX 19 kb)
