# Supplementary material for: Variation and ethnic inequalities in treatment of common mental disorders before, during and after pregnancy: combined analysis of routine and research data in the Born in Bradford cohort
Source: BMC Psychiatry. 2016 Apr 12;16:99. doi: 10.1186/s12888-016-0805-x (PMC4830046; doi:10.1186/s12888-016-0805-x)
Supplement: Additional file 1: Table S1. — List of drug prescriptions and Read codes. (DOCX 19 kb) [file 12888_2016_805_MOESM1_ESM.docx]

**Supplementary material**

**Table S1. List of drug prescriptions and Read codes**

| Indication | Classification / action | List |
| --- | --- | --- |
| **Drugs** |  |  |
| Drugs used to treat CMD | Identified  Treated Pharmacologically | agomelatine, alprazolam, alventa, alventa xl, angilol, ativan, bonilux, bonilux xl, buspironehydrochloride, chloralbetaine, chloralhydrate, chloralmixture, bp2000, cipralex, cipramil, circadin, citalopram, clomipramine, clomipraminehydrochloride, clonezapam, depefex, depefex xl, diazepam, dosulepin, dosulepinhydrochloride, edronax, efexor, efexor xl, escitalopram, faverinparoxetine, feprapax, fluoxetine, flurazepam, fluvoxamine, fluvoxaminemaleate, foraven, foraven xl, gamanil, hloralhydrate, imipramine, imipraminehydrochloride, isocarboxazid, lofepramine, lomont, loprazolam, lorazepam, lormetazepam, lustral, manerix, marplan, melatonin, meprobamatemeprobamate, mianserin, mianserinhydrochloride, mirtazapine, moclobemide, molipaxin, nardil, nitrazepam, optimax, oxactin, oxazepam, parnate, paroxetine, phenelzine, politid, politid xl, propranolol, propranololhydrochloride, prothiaden, prozac, ranfaxine, ranfaxine xl, reboxetine, seroxat, sertraline, sonata, stilnoct, surmontil, syprol, temazepam, tifaxin, tifaxin xl, tranylcypromine, trazodone, trazodonehydrochloride, trimipramine, tryptophan, valdoxan, venaxx, venaxx xl, venlafaxine, venlafaxine m/r, vensir, vensir xl, welldorm, winfex, winfex xl, zaleplon, zimovane, zispin, zispinsoltab, zolpidem, zolpidemtartrate, zopiclone. |
| Drugs used to treat SMI | Cases dropped | abilify, acuphase, amisulpride, anquil, aripiprazole, asenapine, benperidol, camcolit, clopixol, clopixol, clozapine, clozaril, denzapine, depixol, depixol conc., depixol low volume, dolmatil, fluanxol, flupentixol, flupentixol decanoate, fluphenazine decanoate, haldol decanoate, invega, li-liquid, liskonum, lithium carbonate, lithium citrate, modecate, modecate concentrate, olanzapine, olanzapine embonate, orap, paliperidone, pericyazine, pimozide, piportil depot, pipotiazine,, palmitate, priadel, promazine, promazine hydrochloride, quetiapine, risperdal, risperdal consta, risperidone, seroquel, seroquel xl, solian, sulpiride, sulpor, sycrest, xeplion, zaponex, zuclopenthixol, zuclopenthixol acetate, zuclopenthixol decanoate, zypadhera, zyprexa. |
| Drugs used to treat SMI and non-mental health conditions | Cases dropped | carbamazepine, chlorpromazine, chlorpromazine hydrochloride , convulex, depakote, dozic, fentazin, haldol, haloperidol, largactil, levomepromazine, nozinan, perphenazine, serenace, stelazine, tegretol trifluoperazine, valproic acid. |
| **Read codes** |  |  |
| Depression | Identified  Identified as having depression | 1B17., 1B19., 1B1U., 2257., 62T1., E112., E1120, E1121, E1122, E1123, E1125, E1126, E112z, E113., E1130, E1131, E1132, E1135, E1136, E1137, E113z, E118., E11y2, E11z0, E11z1, E11zz, E204., E210., E211., E2110, E2112, E2B.., E2B0., E2B1., Eu320, Eu321, Eu322, Eu324, Eu325, Eu326, Eu327, Eu32B, Eu32y, Eu32z, Eu330, Eu331, Eu33y, Eu33z, Eu34., Eu340, Eu34y, Eu34z, Eu3y., Eu3y1, Eu3yy, Eu3z., Eu53., Eu530, X00SO, X00SR, X00SS, X00SU, X00TX, X40Dl, X40Dm, X760u, X7617, X761I, X761J, X761K, X761L, XE0re, XE0uv, XE1Xy, XE1Y0, XE1Y1, XE1YC, XE1ZY, XE1Za, XE1Zb, XE1Zc, XE1Zd, XE1Zf, XE1Zg, XE1Zh, XE1Zi, XE1aY, XE1ae, XM0Ar, XM0CR, XM1GC, XSEGJ, XSGok, XSGol, XSGom, Xa02E, Xa0wV, Xa110, Xa17z, Xa1eL, Xa9E0, Xa9J0, Xa9K0, XaCHr, XaCHs, XaCIs, XaCIt, XaCIu, XaImU, XaJWh, XaKUk, XaPKm, XaPOv, XaX0C, XaY2C, XaAyL, XaB5v, XaB95, XaB9J |
| Anxiety | Identified  Identified as having anxiety | 1B13., 1B1V., 2258., 225J., E0300, E0310, E200., E2000, E2001, E2002, E2004, E2005, E200z, E201., E2010, E2011, E2012, E2013, E2014, E2015, E2016, E2017, E2018, E201A, E201B, E201C, E201z, E202., E2020, E2021, E2022, E2023, E2024, E2025, E2026, E2027, E2028, E2029, E202A, E202B, E202C, E202D, E202E, E202z, E203., E2030, E2031, E203z, E205., E207., E20y., E20y0, E20y1, E20y2, E20y3, E20yz, E20z., E28.., E280., E281., E282., E283., E2830, E2831, E283z, E284., E28z., Eu40., Eu400, Eu401, Eu402, Eu40y, Eu40z, Eu41., Eu410, Eu411, Eu41y, Eu41z, Eu42., Eu420, Eu421, Eu422, Eu42y, Eu42z, Eu515, Eu51y, Eu51z, Ub1T9, X00Sc, X00Sf, X761N, XE0rb, XE1Y7, XE1YA, XE1Ym, XE1Yn, XE1Zj, XE1aW, XE1bo, XM1MZ, Xa0XG, Xa0XH, Xa0XI, Xa0XJ, Xa0XK, Xa0XM, Xa0XN, Xa0XO, Xa0XP, Xa0XQ, Xa0XR, Xa0XX, Xa0XY, Xa0Xd, Xa18j, Xa18v, Xa19B, Xa3Xk, Xa3Ys, Xa7kB, XaEFB, XaP8d, XaX55, XaX56, XaX58 |
| Comorbid depression & anxiety | Identified | E2003, Eu412, Eu413, X00Sb |
| CMD treatment or referral for CMD treatment , or CMD-related follow-up | Identified  Treated non-pharmacologically | 6655., 6659., 66590, 6779., 6G00., 8BK0., 8BM0., 8CQ.., 8CR7., 8F85., 8G..., 8G1.., 8G10., 8G100, 8G11., 8G12., 8G120, 8G121, 8G2.., 8G21., 8G2Z., 8G4.., 8G43., 8G4Z., 8G5.., 8G51., 8G5Z., 8G6.., 8G6Z., 8G7.., 8G7Z., 8G9.., 8G91., 8G9Z., 8HlB., 8HVO., 8H23., 8H230, 8H34., 8H38., 8H49., 8H7A., 8H7B., 8H7T., 8H7Z., 8HHp., 8HHq., 8HJ3., 8HK9., 8HkK., 8HM9., 9HZ.., 9N0T., 9N1M., 9N2B., 9N6h., 9NJ1., 9NJR., 9NJT., 9Ol.., Ub0qs, X71Ec, X71bp, X79sL, XE0iL, XE1Sa, XE1Sb, XSBbs, Xa8IB, Xa8IG, Xa8IJ, Xa8IP, Xa8IR, Xa8If, Xa8Ig, Xa8Ih, Xa8Ii, Xa8Ij, Xa8Ik, Xa8Is, Xa8It, Xa8Iu, Xa8Ix, Xa8J0, XaA8Z, XaA8c, XaA8d, XaA8u, XaA8v, XaA9W, XaA9g, XaABP, XaABQ, XaAKy, XaAMj, XaAMz, XaAOd, XaAOe, XaAOf, XaAOg, XaAOh, XaAQi, XaAQo, XaAS4, XaAU5, XaAUA, XaAXe, XaAZI, XaAbC, XaAbH, XaAdM, XaAel, XaAem, XaAen, XaAfJ, XaAh4, XaAiE, XaAiI, XaAkB, XaAkI, XaAkU, XaAnb, XaBHK, XaBIg, XaBJb, XaBJc, XaBT1, XaBTD, XaBtN, XaBvV, XaBvW, XaBvX, XaCFD, XaECG, XaEVq, XaI8j, XaINQ, XaINy, XaIOf, XaIOg, XaIOh, XaIOi, XaIOj, XaIOk, XaIOl, XaIOn, XaIOp, XaIOq, XaIOs, XaIOu, XaIOv, XaIOy, XaIOz, XaIP0, XaIP1, XaIP2, XaIP3, XaIPw, XaISp, XaISv, XaISw, XaISy, XaIT1, XaIT2, XaIT3, XaIT4, XaIT5, XaIT6, XaIT7, XaIT8, XaITA, XaITG, XaITH, XaITI, XaIUv, XaIUx, XaIUy, XaIUz, XaIV0, XaIV1, XaIV2, XaIV3, XaIV4, XaIV5, XaIV6, XaIW3, XaIW4, XaIW5, XaIW6, XaIWD, XaIWM, XaIWN, XaIWR, XaIWS, XaIWT, XaIWU, XaIWV, XaIWW, XaIWX, XaIWY, XaIWZ, XaIWa, XaIWb, XaIWx, XaIWy, XaIWz, XaIX0, XaIXS, XaIXT, XaIXU, XaIXV, XaIXW, XaIXX, XaIXY, XaIXZ, XaIXa, XaIXb, XaIXh, XaIXi, XaIXk, XaIXl, XaIXm, XaIXn, XaIXo, XaIXp, XaIXq, XaIXs, XaIXt, XaIXu, XaIYN, XaIkd, XaIkg, XaIku, XaIm4, XaIpA, XaItc, XaItx, XaIuR, XaIvk, XaIvp, XaIvq, XaIyU, XaJ4V, XaJ4w, XaJ4x, XaJOA, XaJON, XaJPu, XaJPz, XaJQ1, XaJQD, XaJQE, XaJQF, XaJQG, XaJQH, XaJQI, XaJQJ, XaJQR, XaJQS, XaJQT, XaJQU, XaJQV, XaJQW, XaJQX, XaJQY, XaJQZ, XaJRr, XaJWg, XaJr3, XaK1f, XaK5q, XaK5r, XaK6K, XaK70, XaK71, XaKAx, XaKEz, XaKGq, XaKbb, XaL03, XaL0o, XaL0p, XaL0q, XaL0r, XaL0s, XaL0t, XaL0u, XaL0v, XaL0w, XaL2L, XaLBl, XaLCP, XaLCQ, XaLFL, XaLFk, XaLNF, XaLQw, XaLnp, XaLnq, XaLnr, XaLst, XaLsu, XaLsv, XaM2K, XaM7s, XaMGz, XaMJ8, XaMhM, XaN3a, XaN4b, XaN4c, XaN4d, XaN4e, XaN4f, XaN4g, XaNPL, XaNTc, XaONq, XaOOT, XaObo, XaOxM, XaP6T, XaP7x, XaPRF, XaPTT, XaPTU, XaPlZ, XaPvy, XaPvw, XaQBz, XaQC0, XaQWJ, XaQvz, XaR4n, XaR4s, XaR5D, XaWzW, XaX04, XaXEJ, XaXH8, XaXHm, XaXe3, XaXiH, XaXl2, XaY6o, XaY7i, XaYgS, XaZIW, XaZcf, ZV663, ZV673, ZV69., ZV690, ZV691, ZV692, ZV6D., ZV701, ZV702, 665.., 6654., 6658., 66580, 665A., 665A0, 665Z., 8A2.., 8A21., 8A2Z., 9H90., 9H91., 9H92., 9HA0., 9Ov.., 9Ov0., 9Ov1., 9Ov2., 9Ov3., 9Ov4., X74WN, XaJuG, XaJuK, XaJuT, XaJuV, XaJuW, XaK6d, XaK6e, XaK6f, XaK9p, XaKAK, XaLIb, XaMGL, XaMGN, XaMGO, XaMGP, XaMGQ, XaMGR, XaR9y, XaZ2p |
| SMI | Cases dropped | 1B1b., 225E., 225F., 6656., 6657., 665B., 665C., 665D., 665E., 665F., 665G., 665H., 665J., 665K., 8HHs., 9H1.., 9H11., 9H12., 9H13., 9H14., 9H1Z., 9H2.., 9H21., 9H22., 9H23., 9H24., 9H25., 9H2Z., 9H3.., 9H31., 9H32., 9H33., 9H34., 9H3Z., 9H4.., 9H41., 9H42., 9H43., 9H44., 9H45., 9H4Z., 9H5.., 9H51., 9H52., 9H53., 9H54., 9H55., 9H5Z., 9H7.., 9H8.., 9Ol6., 9Ol7., E10.., E100., E1000, E1001, E1002, E1003, E1004, E1005, E100z, E101., E1010, E1011, E1012, E1013, E1014, E1015, E101z, E102., E1020, E1021, E1022, E1023, E1024, E1025, E102z, E103., E1030, E1031, E1032, E1033, E1034, E1035, E103z, E104., E110., E1100, E1101, E1102, E1103, E1104, E1105, E1106, E110z, E111., E1110, E1111, E1112, E1113, E1114, E1115, E1116, E111z, E1124, E1133, E1134, E114., E1140, E1141, E1142, E1143, E1144, E1145, E1146, E114z, E115., E1150, E1151, E1152, E1153, E1154, E1155, E1156, E115z, E116., E1160, E1161, E1162, E1163, E1164, E1165, E1166, E116z, E117., E1170, E1171, E1172, E1173, E1174, E1175, E1176, E117z, E11y., E11y0, E11y1, E11y3, E11yz, E11z., E12.., E120., E121., E122., E123., E12y., E12y0, E12yz, E12z., E13.., E130., E131., E132., E133., E134., E135., E13y., E13y0, E13y1, E13yz, E13z., E14.., E141., E1411, E141z, E14y., E14y1, E14yz, E14z., E1y.., E1z.., E21.., E2111, E2112, E2113, E211z, E212., E2120, E2121, E2122, E212z, E213., E214., E2140, E2141, E214z, E215., E2150, E2151, E2152, E2153, E215z, E216., E217., E21y., E21y1, E21y2, E21y3, E21y4, E21y5, E21y6, E21y7, E21yz, E21z., Eu1.., Eu10., Eu100, Eu101, Eu102, Eu103, Eu104, Eu105, Eu106, Eu107, Eu108, Eu10y, Eu10z, Eu11., Eu110, Eu111, Eu112, Eu113, Eu114, Eu115, Eu116, Eu117, Eu11y, Eu11z, Eu12., Eu120, Eu121, Eu122, Eu123, Eu124, Eu125, Eu126, Eu127, Eu12y, Eu12z, Eu13., Eu130, Eu131, Eu132, Eu133, Eu134, Eu135, Eu136, Eu137, Eu13y, Eu13z, Eu14., Eu140, Eu141, Eu142, Eu143, Eu144, Eu145, Eu146, Eu147, Eu14y, Eu14z, Eu15., Eu150, Eu151, Eu152, Eu153, Eu154, Eu155, Eu156, Eu157, Eu15y, Eu15z, Eu16., Eu160, Eu161, Eu162, Eu163, Eu164, Eu165, Eu166, Eu167, Eu16y, Eu16z, Eu17., Eu170, Eu171, Eu172, Eu173, Eu174, Eu175, Eu176, Eu177, Eu17y, Eu17z, Eu18., Eu180, Eu181, Eu182, Eu183, Eu184, Eu185, Eu186, Eu187, Eu18y, Eu18z, Eu19., Eu190, Eu191, Eu192, Eu193, Eu194, Eu195, Eu196, Eu197, Eu19y, Eu19z, Eu1A., Eu1A0, Eu1A1, Eu1A2, Eu1A3, Eu1A4, Eu1A5, Eu1A6, Eu1A7, Eu1Ay, Eu1Az, Eu2.., Eu20., Eu200, Eu201, Eu202, Eu203, Eu204, Eu205, Eu206, Eu20y, Eu20z, Eu21., Eu22., Eu220, Eu221, Eu222, Eu223, Eu22y, Eu22z, Eu23., Eu230, Eu231, Eu232, Eu233, Eu23y, Eu23z, Eu24., Eu25., Eu250, Eu251, Eu252, Eu25y, Eu25z, Eu26., Eu2y., Eu2z., Eu30., Eu300, Eu301, Eu302, Eu30y, Eu30z, Eu31., Eu310, Eu311, Eu312, Eu313, Eu314, Eu315, Eu316, Eu317, Eu318, Eu319, Eu31y, Eu31z, Eu323, Eu328, Eu329, Eu32A, Eu333, Eu341, Eu3z., Eu44., Eu440, Eu441, Eu442, Eu443, Eu444, Eu445, Eu446, Eu447, Eu44y, Eu44z, Eu45., Eu450, Eu451, Eu452, Eu453, Eu454, Eu455, Eu45y, Eu45z, Eu46., Eu460, Eu461, Eu46y, Eu46z, Eu5.., Eu531, Eu54., Eu55., Eu5z., Eu6.., Eu61., Eu60., Eu601, Eu602, Eu603, Eu604, Eu605, Eu606, Eu607, Eu608, Eu60y, Eu60z, Eu62., Eu620, Eu621, Eu62y, Eu62z, Ua1WW, Ub1T7, X73gl, X73gm, X73gn, X73go, X75yp, X75yv, X75yw, X75z5, X75z7, X75zA, X75zC, X75zE, X761M, X79ul, XE1Xt, XE1Xw, XE1Xx, XE1Y2, XE1Y3, XE1Y4, XE1Y5, XE1Y6, XE1YF, XE1YG, XE1YH, XE1YI, XE1YJ, XE1YK, XE1YL, XE1YM, XE1ZM, XE1ZN, XE1ZO, XE1ZP, XE1ZQ, XE1ZR, XE1ZU, XE1ZX, XE1ZZ, XE1Ze, XE1Zy, XE1aM, XE1aO, XE1aQ, XE1aS, XE1aU, XE1ag, XE1am, XE1gG, XE1ic, XE1ji, XE2RN, XE2b6, XE2b8, XE2uT, XE2un, XE2v2, XM1GG, XM1Yd, XSGon, Xa1aD, Xa1hV, Xa3WO, Xa3Xd, Xa3Xf, Xa3Xg, Xa3Y9, Xa3aC, Xa3aF, Xa3aL, Xa3aP, Xa3aU, Xa3aV, Xa3aW, Xa3aX, Xa3at, Xa4HV, Xa4Ha, Xa8Nf, Xa8O2, Xa8OA, Xa8OE, Xa8Og, Xa8Oh, Xa8Oi, Xa8Pk, Xa8Qw, Xa8Qx, Xa8Qy, Xa8Qz, Xa9Dh, Xa9Di, Xa9Dk, Xa9Dm, Xa9Do, Xa9Dr, Xa9Ds, Xa9Dt, Xa9Du, Xa9Dv, Xa9Dw, Xa9Dx, Xa9Dy, Xa9Dz, Xa9E1, Xa9E2, Xa9EC, Xa9EE, Xa9EF, Xa9EG, Xa9EI, Xa9EM, Xa9EP, Xa9EQ, Xa9ER, Xa9EV, Xa9EW, Xa9EX, Xa9EY, Xa9EZ, Xa9Ea, Xa9Eb, Xa9Ec, Xa9Ed, Xa9Ee, Xa9Ef, Xa9Eg, Xa9Ei, Xa9Ej, Xa9Ek, Xa9El, Xa9Em, Xa9Eo, Xa9Ep, Xa9Eq, Xa9Er, Xa9Es, Xa9Et, Xa9Eu, Xa9Ev, Xa9GR, Xa9GU, Xa9GV, Xa9GX, Xa9GZ, Xa9Gc, Xa9IW, Xa9IX, Xa9IY, Xa9IZ, Xa9Ib, Xa9Ic, Xa9Id, Xa9Ie, Xa9If, Xa9Ig, Xa9Ih, Xa9Ii, Xa9Ij, Xa9Ik, Xa9Il, Xa9Im, Xa9In, Xa9Ip, Xa9Iq, Xa9Is, Xa9Iw, Xa9Iy, Xa9J3, Xa9J4, Xa9J5, Xa9J6, Xa9JA, Xa9JC, Xa9JE, Xa9JG, Xa9JH, Xa9JJ, Xa9JK, Xa9JR, Xa9Ja, Xa9Jb, Xa9Jd, Xa9Jf, Xa9Jh, Xa9Jj, Xa9Jl, Xa9Jn, Xa9Jp, Xa9Jq, Xa9Jr, Xa9Js, Xa9Jt, Xa9Jz, Xa9K2, Xa9K3, Xa9K4, Xa9K5, XaA6j, XaA6x, XaA9j, XaA9r, XaA9s, XaBHL, XaBHM, XaBHN, XaBHO, XaBYV, XaBYW, XaBYX, XaBYY, XaBYZ, XaBhM, XaCHo, XaIOm, XaIWE, XaIWF, XaIWG, XaIWH, XaIWI, XaIWJ, XaIWK, XaIWL, XaIXj, XaJQO, XaKUl, XaKUm, XaL19, XaLIa, XaMwc, XaMwd, XaMwe, XaNlN, XaPYK, XaPYL, XaX51, XaX52, XaX53, XaX54, XaY1Y |

Case-free searching employed for drugs; CMD common mental disorder; SMI severe mental illness
